# Three-Dimensional Bioprinting: A Comprehensive Review for Applications in Tissue Engineering and Regenerative Medicine

**DOI:** 10.3390/bioengineering11080777

**Published:** 2024-07-31

**Authors:** Nicholas A. Mirsky, Quinn T. Ehlen, Jason A. Greenfield, Michael Antonietti, Blaire V. Slavin, Vasudev Vivekanand Nayak, Daniel Pelaez, David T. Tse, Lukasz Witek, Sylvia Daunert, Paulo G. Coelho

**Affiliations:** 1 University of Miami Miller School of Medicine, Miami, FL 33136, USA; 2 Department of Biochemistry and Molecular Biology, University of Miami Miller School of Medicine, Miami, FL 33136, USA; 3 Dr. Nasser Ibrahim Al-Rashid Orbital Vision Research Center, Bascom Palmer Eye Institute, University of Miami Miller School of Medicine, Miami, FL 33136, USA; 4 Biomaterials Division, NYU Dentistry, New York, NY 10010, USA; 5 Department of Biomedical Engineering, New York University Tandon School of Engineering, Brooklyn, NY 11201, USA; 6 Hansjörg Wyss Department of Plastic Surgery, New York University Grossman School of Medicine, New York, NY 10016, USA; 7 DeWitt Daughtry Family Department of Surgery, Division of Plastic Surgery, University of Miami Miller School of Medicine, Miami, FL 33136, USA

**Keywords:** 3D bioprinting, regenerative medicine, tissue engineering, surgical implants, bioinks, orthopedic surgery, plastic and reconstructive surgery, ophthalmology

## Abstract

Since three-dimensional (3D) bioprinting has emerged, it has continuously to evolved as a revolutionary technology in surgery, offering new paradigms for reconstructive and regenerative medical applications. This review highlights the integration of 3D printing, specifically bioprinting, across several surgical disciplines over the last five years. The methods employed encompass a review of recent literature focusing on innovations and applications of 3D-bioprinted tissues and/or organs. The findings reveal significant advances in the creation of complex, customized, multi-tissue constructs that mimic natural tissue characteristics, which are crucial for surgical interventions and patient-specific treatments. Despite the technological advances, the paper introduces and discusses several challenges that remain, such as the vascularization of bioprinted tissues, integration with the host tissue, and the long-term viability of bioprinted organs. The review concludes that while 3D bioprinting holds substantial promise for transforming surgical practices and enhancing patient outcomes, ongoing research, development, and a clear regulatory framework are essential to fully realize potential future clinical applications.

## 1. Introduction

Three-dimensional (3D) printing, also known as additive manufacturing (AM), is a revolutionary “bottom-up” manufacturing technique used to fabricate customized physical objects (devices) and bulk products [1]. Since its introduction in the early 1980s, 3D printing technology has experienced rapid progress. Consequently, the advancement of affordable 3D printing equipment and the availability of open-source software have greatly enhanced accessibility. The rising popularity of 3D printing over the past two decades in different industries, including the aerospace, automotive, dental, and medical industries, can be attributed to its versatility, relative ease of use, and capacity to manufacture complex shapes and structures. It is worth mentioning that the medical industry has been slow to adopt the usage of 3D printing relative to other industries, partly owing to strict standards including in vivo performance and safety prior to market availability. Nonetheless, advancements in imaging technology and materials science have paved the way for the effective use of 3D printing in modern medicine. Three-dimensional printing not only circumvents disadvantages associated with conventional, subtractive, manufacturing modalities by allowing for the fabrication of complex geometries and heterogenous constructs on an individual level. It also alleviates the need for conventional grafting techniques that often rely upon the harvesting of tissue from a healthy donor site, namely, autografting—a technique limited by donor site morbidity, finite tissue, prolonged operative times, and significant post-operative pain. Currently, computer-aided design (CAD) in conjunction with computed tomography (CT) and magnetic resonance imaging (MRI) scans has allowed investigators to model 3D-printed constructs based on patient-specific imaging, creating implants that can be used for surgical guidance and the reconstruction of a variety of defects [2].

Researchers have also used principles of this technique to develop 3D bioprinting technology, which involves the layer-by-layer deposition of bioinks—substances composed of living cells, biomaterials, and active biomolecules—to fabricated structures that closely mimic native tissues [3]. In essence, 3D bioprinting allows for the creation of complex structures and spatial heterogeneities that closely resemble the natural structure of the tissue being replicated. To elaborate, the ability to spatially control the nano-, micro-, and macrostructures of cell-laden constructs may replicate complex native tissue architecture more reliably than traditional tissue engineering methods [4]. If successful, this technique could allow for the manufacturing of physiologically relevant heterogenous tissue constructs on demand, alleviating the aforementioned need for autologous tissue harvesting and potentially transforming surgical treatments across various disciplines [5,6].

Research in 3D bioprinting has demonstrated significant translational potential across different surgical fields such as plastic and reconstructive surgery, orthopedics, and ophthalmology [7,8,9]. In plastic and reconstructive surgery, it has enabled the creation of skin grafts and complex tissue structures for cosmetic and functional restoration [7]. Orthopedics has benefited from the ability to print bone grafts and cartilage, potentially reducing the need for invasive grafting/harvesting procedures [8]. In ophthalmology, researchers are exploring bioprinted tissues that could one day restore vision or repair eye injuries [9]. These few examples of applications highlight the diverse potential of bioprinting technologies; however, these applications are not without challenges. There remain fundamental scientific questions yet to be answered prior to implementation in the clinical space [10].

Issues such as the vascularization of printed tissues, integration with host tissues, and the long-term viability and functionality of bioprinted organs remain significant hurdles [11]. Furthermore, the replication of biological tissues at the microscale is frequently required. Therefore, it is necessary to fully understand the microenvironment and composition of the extracellular matrix (ECM), and physiological forces at play. Moreover, ethical considerations regarding the source and use of cellular materials in bioprinting require careful deliberation and regulatory oversight to ensure that these innovations benefit society without compromising on moral or ethical standards [12].

The goal of this review is to highlight recent developments and advances in 3D bioprinting within the context of surgical applications. It aims to showcase the transformative potential of this technology, discuss its limitations, and explore future directions that could further revolutionize the field. By providing a comprehensive overview of how 3D bioprinting is being integrated into various surgical specialties, this paper seeks to inspire and guide ongoing research and underscore the critical role of 3D bioprinting in the future of regenerative medicine.

## 2. Methods

A literature search was conducted using the PubMed electronic database in May 2024. Key terms, Medical Subject Headings (MeSH) terms, and Boolean operators (‘AND’ and ‘OR’) were used in the database search to create three individual search queries for each of the specialties reviewed (Plastic and Reconstructive Surgery, Orthopedic Surgery, and Ophthalmology). Search terms used across the three queries included ‘3D bioprinting’, ‘3D bioprinted’, and ‘bioinks’. Search queries used for each specialty search were as follows:

Plastic and Reconstructive Surgery: (“3D bioprinting” OR “3D bioprinted” OR “bioinks”) AND (“Plastic surgery” OR “Reconstructive surgery”).

Orthopedic Surgery: (“3D bioprinting” OR “3D bioprinted” OR “bioinks”) AND (“Orthopedics” OR “Orthopedics” OR “bone” OR “cartilage” OR “tendon” OR “ligament” OR “musculoskeletal”).

Ophthalmology: (“3D bioprinting” OR “3D bioprinted” OR “bioinks”) AND ((“Ophthalmology” OR “ocular” OR “eye”) OR (“oculoplastic” OR “orbit” OR “eyelid” OR “tear duct”)).

Given that 3D bioprinting is a rapidly evolving technology, the search was restricted to articles published within the last five years (2019–2024). Articles were only included if they were original, full-text, in the English language, and involved in vitro and/or in vivo study designs. Reports were excluded if they (1) did not involve 3D bioprinting or (2) were not relevant to the searched specialty. Additionally, any review articles, case reports, case series, editorials, book chapters, erratum, and abstracts with no detailed report of their results were excluded. Articles identified were managed in EndNote and any duplicates removed. After screening, sixty-nine plastic and reconstructive surgery, seventy-six orthopedic, and forty-five ophthalmology articles were included in their respective specialty-specific discussions.

## 3. Bioprinting in Plastic and Reconstructive Surgery

Plastic and reconstructive surgery represents a cornerstone of bioprinting’s rapid technological innovation. The goal of restoring form and function across the entire body requires a wide variety of tissue types for defect reconstruction, including dermal, neural, and adipose tissues. However, there remains a significant lack of donor tissues in the United States, which has served as an impetus for bioprinting research [13]. In this section, we explore recent developments in bioprinting’s application within plastic and reconstructive surgery, highlighting both representative in vitro and in vivo studies as well as challenges to translation (Table 1).

### 3.1. Skin and Wound Healing

The human skin consists of a complex arrangement of various sensory neurons, blood vessels, hair follicles, and sweat glands performing a variety of functions such as protection and the transfer of information [24]. Their structural integrity remains inherently delicate, despite skin’s strong regenerative capacity. Skin defects due to trauma, burns, non-healing ulcers, and chronic infections commonly do not heal on their own, necessitating autologous skin grafts for proper rehabilitation [25]. Autologous grafts are not without significant drawbacks including scar proliferation, secondary infections, and a lack of native structural complexity after healing [26].

The bioprinting of lab-engineered skin attempts to mitigate respective drawbacks. Three-dimensional printing fabrication offers the advantage of the controlled spatial distribution of cells and manipulation of scaffolds to mimic the complex structure of native skin [24]. Most approaches utilize a combination of keratinocytes and fibroblasts, attempting to mimic the epidermal and dermal layers, respectively [27]. The layer-by-layer 3D printing approach offers precise constructs for subsequent implantation [28]. However, bioprinting on planar substrates for subsequent deployment onto target surfaces can lead to geometrically mismatched interfaces in cases of irregular wound contours, making implantation and successful integration challenging [29]. Wu et al. introduced a curvilinear bioprintable gelatin/polyurethane bioink laden with keratinocytes, fibroblasts, and endothelial progenitor cells, implanted in a rodent model of chronic and irregular wounds [14]. This curvilinear model was fabricated based on individual rodent wound topography and demonstrated full reepithelization and dermal repair, as well as substantial amounts of neovascularization and collagen production after 28 days [14].

An additional and common challenge researchers encounter when developing skin tissue substitutes is the ease of fabrication as biomaterials/additives of interest may be limited and require complex preparation [30]. An additive of growing interest includes the decellularized extracellular matrix (dECM), which is capable of mimicking the natural microenvironment of regenerating skin [31]. Zhang et al. developed a simplified and rapid method to create a bioink using microfragmented adipose extracellular matrix (mFAECM), adipose tissue that was previously non-extrudable from 3D bioprinters [15]. The in vivo and in vitro outcomes of this study demonstrated the feasibility of creating a mFAECM bioink, accelerating wound healing via the promotion of collagen secretion, remodeling, and neovascularization [15]. Other components of adipose tissue under investigation include adipose-derived mesenchymal stem cells (ADSCs), which have been proven to reduce scar formation and increase collagen type III deposition in burn wound healing. However, in vivo studies have failed to demonstrate adequate cell viability and efficiency after grafting [32,33]. Nitric oxide (NO) has recently become an attractive candidate to improve the bioactivity of ADSCs in scaffold placement by regulating inflammation and angiogenesis [34,35]. Wu Y et al. investigated the addition of NO to 3D-bioprinted ADSCs hydrogel scaffolds, demonstrating enhanced burn wound healing in mice by promoting rapid epithelialization, increased collagen deposition, and neovascularization [16]. This study suggested a promising treatment strategy that leverages both the regenerative potential of ADSCs and the therapeutic effects of NO in severe burn management [16].

Another major challenge involves the management and prevention of infected wounds due to the risk of bacterial resistance and inefficacy of conventional wound dressings [36,37]. Photodynamic therapy (PDT) has garnered widespread attention in preventing wound infections and avoiding antibiotic use due to its ability to induce bacterial toxicity under the action of specific light wavelengths [38]. Li et al. developed a novel 3D-bioprinted hydrogel incorporated with methylene blue nanoparticles, designed to provide PDT antibacterial action tested in vitro and in vivo [17]. The outcomes of this study demonstrated significant reduction in viability of common wound pathogens in vitro while also improving full-thickness skin defect repair in mice [17]. The significance of these studies demonstrates 3D bioprinting’s application not only in skin substitute development but also in wound dressing and infection control.

### 3.2. Epidermal Appendages

#### 3.2.1. Melanocytes

**Figure 1:**
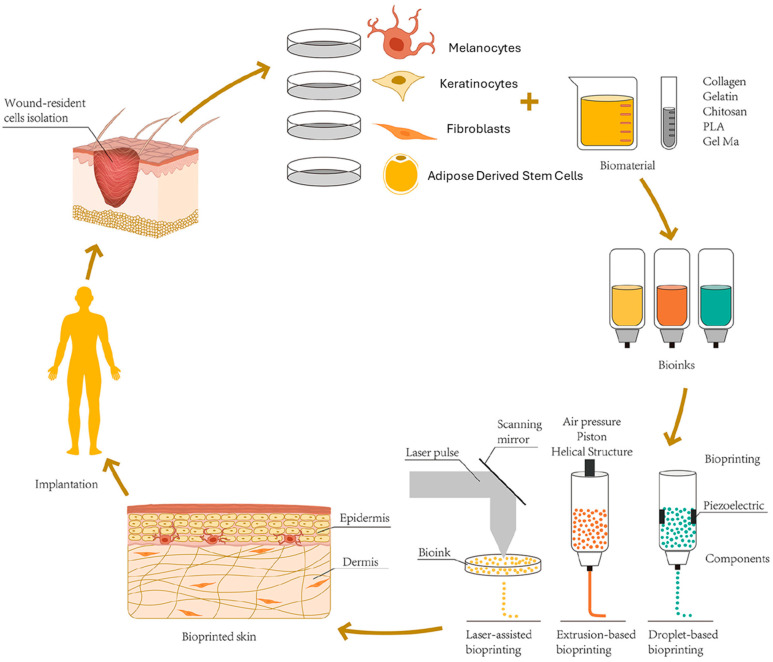
Schematic representation of skin 3D bioprinting. Reprinted (adapted) from Weng et al. [24] under the terms of the Creative Commons Attribution—Noncommercial 4.0 License. https://creativecommons.org/licenses/by-nc/4.0/ (accessed on 11 July 2024).

The skin’s complex architecture is intimately associated with epidermal appendages such as hair follicles, sweat glands, and melanocytes, which present a significant hurdle in bioprinting (Figure 1). These appendages have extremely poor regenerative capacity due to unique microvascular networks, making them challenging to regenerate after congenital diseases, severe burns, and trauma [39,40]. Currently, there is no established skin implant that incorporates fully functional melanocytes [41]. Furthermore, only two of the studies from our literature search attempted to develop a full-thickness skin implant with the incorporation of melanocytes [42,43]. Min et al. attempted to incorporate melanocytes into their full-thickness bioprinted in vivo skin model, leading to freckled pigmentation of the implanted graft at the dermal–epidermal junction with no exposure to external ultraviolet light [43]. In addition, Jorgensen et al. demonstrated that bioprinting could precisely fabricate complex tissue structures including skin with melanocytes and other epidermal appendages [42]. Their study showed that bioprinted skin-treated wounds had complete closure and normal collagen remodeling in in vivo mouse models; however, there was no notable pigmentation in the treated wound areas grossly [42]. While these results demonstrated melanocyte incorporation and viability of bioprinted skin, further studies are required to optimize and to fully mimic the native functionality of skin. Furthermore, upcoming research could delve into examining the potential of 3D-bioprinted constructs with exposure to external stimuli to conclude this model’s translational potential prior to trials in humans.

#### 3.2.2. Hair Follicles and Sweat Glands

Other important epidermal appendages such as hair follicles and sweat glands are normally found in the reticular dermis of the skin. These two appendages rely on the stem-cell like units (i.e., dermal papilla cells (DPCs) for hair follicles) of native skin to cyclically regenerate. These units play key roles in wound regeneration and the protection of underlying structures [44]. Patients with extensive trauma and deep burns are often unable to dissipate heat through sweat and regenerate native hair patterns, representing a highly influential area of continued research. Commercially available cell lines for these structures are available; however, the majority of preclinical studies focused on full-thickness skin regeneration have not attempted to incorporate these native appendages [45]. In terms of bioprinting hair follicles, Zhang et al. was able to establish an in vitro model where 3D-bioprinted porous scaffolds contained sweat gland cells that were seeded with hair follicle units. They observed a symbiotic relationship between the two appendages wherein only in the presence of both structures were the cells able to fully regenerate [46]. Furthermore, Kang et al. developed a 3D-bioprinted, multi-layered scaffold for DPCs, representing one of the few in vivo studies on this topic. Each layer of the scaffold incorporated fibroblasts, epidermal cells, and DPCs, encapsulated in a gelatin–alginate hydrogel that effectively simulated the microenvironment required for hair follicle regeneration [47]. In terms of sweat glands, some studies have attempted to regenerate these structures and understand their complex biomimetic microenvironment in vitro [48,49,50]. Yao et al. employed an extrusion-based bioprinting method to replicate the sweat gland microenvironment in vitro, driving the differentiation of MSCs into functional sweat gland units. This study was able to identify two essential factors that boost sweat gland gene expression profiles, namely Collagen Triple Helix Repeat Containing 1 (CTHRC1), a biochemical regulator for sweat gland specification; and heme oxygenase 1 (*Hmox1*), a gene involved in MSC differentiation [50]. Huang et al. initiatively fabricated a 3D-bioprinted mouse ECM with the purpose of providing an inductive niche for sweat gland regeneration from mouse epidermal progenitor cells. The in vivo transplantation of this construct was performed, resulting in the preliminary restoration of sweat glands in the burned paws of mice [49].

### 3.3. Craniofacial Reconstruction

Equally as complex as human skin repair is the reconstruction of craniofacial defects. Mimicking the complex 3D arrangements and multicellular interactions of craniofacial tissues represents a significant challenge in plastic and reconstructive surgery. Bioprinting has been investigated as a potential solution for navigating the structural arrangement of heterogenous facial tissues; however, its clinical applications are still within the early development stages [51]. Recently, in vivo and in vitro studies investigating auricular, tracheal, and calvaria reconstruction advancements utilizing 3D bioprinting have gained traction in craniofacial surgery.

#### 3.3.1. Auricular and Nasal Reconstruction

Auricular and nasal reconstruction involves the transposition of autologous cartilage grafts with subsequent coverage using autologous skin flaps. In addition to the donor site morbidity of harvested grafts, the sculpting of the cartilage prior to placement is complex, highly skill-dependent, and time-consuming [18]. Ruiz-Cantu et al. targeted this challenge by investigating the use of a 3D bioprinting approach combining chondrocyte-laden gelatin methacrylate and PCL to create a biocompatible, mechanically robust construct design for nasal cartilage repair [18]. The outcomes showed the successful fabrication of a construct that mimicked the mechanical properties of nasal cartilage while simultaneously supporting neocartilage formation [18]. To additionally combat the issue of limited donor skin in auricular reconstruction, Zielinska et al. presented a novel approach using biofabricated auricular grafts that combine bioprinted autologous auricular cartilage with pre-vascularized dermo-epidermal skin substitute [19]. The authors demonstrated the successful integration of these implants in immunocompromised rats, with bioprinted capillaries connecting to the recipient’s vasculature within one week, facilitating rapid perfusion and epidermal maturation [19].

#### 3.3.2. Tracheal Reconstruction

**Figure 2:**
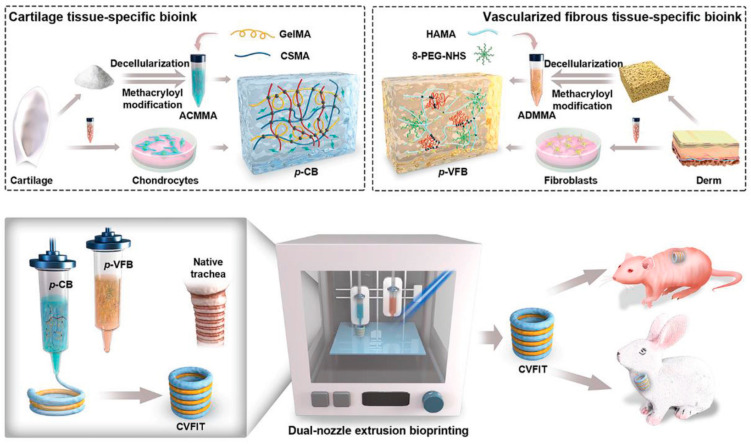
A schematic illustration of instant trachea reconstruction using cartilage- and vascularized fibrous tissue-specific bioinks and 3D-bioprinted cartilage-vascularized fibrous tissue-integrated trachea in mice and in situ trachea reconstruction in rabbits. Abbreviations—ACMMA: methacryloyl-modified acellular cartilage matrix; ADMMA: methacryloyl-modified acellular derm matrix; HAMA: methacrylate-modified hyaluronic acid; 8-PEG-NHS: 8-arm-polyethylene glycol-succinic acid ester; GelMA: gelatin methacryloyl; CSMA: methacrylate-modified chondroitin sulfate; p-CB: photocrosslinkable cartilage-specific bioink; p-VFB: photocrosslinkable vascularized fibrous tissue-specific bioink. Reprinted from Huo et al. [21], under the terms of the Creative Commons Attribution—Noncommercial 4.0 License. https://creativecommons.org/licenses/by/4.0/ (accessed on 11 July 2024).

Three-dimensional bioprinting techniques have continued to emerge as a promising strategy to offer native-like substitutes for complex segmental trachea reconstruction [52]. The trachea comprises a heterogenous structure of alternating cartilage rings connected by vascularized fibrous tissue rings, rendering conventional reconstruction techniques utilizing autologous grafts challenging [53]. Torsello et al. investigated a bioprinted PCL scaffold embedded with autologous mesenchymal stem cells for laryngotracheal reconstruction in an ovine model [20]. The study revealed mixed outcomes wherein two animals showed the complete integration of scaffolds with the growth of the respiratory epithelium on the scaffold’s surface and two animals showed post-operative inflammation and respiratory distress due to the mechanical stiffness of the material. The scaffold developed was not spatially similar to the native trachea and was used as a “patch” for a segmental tracheal defect [20]. On the other hand, Huo et al. proposed a novel strategy utilizing 3D bioprinting to create a cartilage-vascularized fibrous tissue-integrated trachea (CVFIT) structure using photocrosslinkable bioinks that mimic the natural heterogeneity of the trachea (Figure 2) [21]. The in vivo outcomes revealed successful trachea regeneration demonstrating appropriate mechanical properties and physiological functions, such as vascularization and epithelialization [21].

### 3.4. Peripheral Nerve Reconstruction

**Figure 3:**
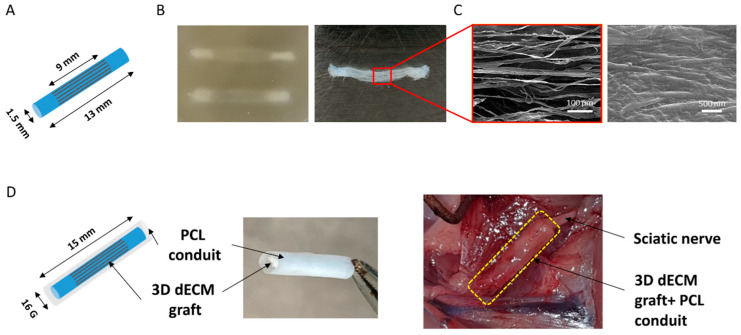
Microgel printing to create highly precise and intricate microfilament structures using dECM bioinks, aiming to mimic the native tissue structures. (**A**) Schematic diagram of the 3D-printed dECM graft. (**B**) Pictograph of the 3D-printed dECM graft in a microgel printing bath and the 3D-printed dECM graft after microgel printing, bath washing, and lyophilization. (**C**) Scanning electron micrographs of the 3D-printed dECM graft at high and low magnifications. (**D**) Schematic of the 3D-printed dECM graft mounted in the PCL conduit and its implantation in vivo. Reprinted (adapted) with permission from Min et al. [23]. Copyright 2022 American Chemical Society.

Nerve injuries represent one of the most challenging tissue types to reconstruct due to their limited regenerative potential. For peripheral nerve injuries, nerve autografts and allografts are currently the standard of care for bridging critical-sized nerve defects [54]. Nevertheless, clinical outcomes remain suboptimal, further complicated by donor site morbidity and the paucity of nerve tissue [54]. To overcome current graft limitations, researchers have been actively developing 3D-bioprinted grafts using various synthetic and decellularized materials to accurately replicative the native nerve structure. Min et al. developed a microgel printing technique for creating highly precise and intricate microfilament structures using decellularized extracellular matrix (dECM) bioinks, aiming to mimic the native tissue structures more closely than conventional bioprinting methods (Figure 3) [23]. Their outcomes demonstrated that the fabricated nerve grafts could effectively support nerve regeneration, achieving results comparable to traditional autologous nerve grafts in a rat sciatic nerve injury model [23]. Recently, growing evidence has demonstrated that nerve guidance conduits (NGCs) combined with post-natal stem cells may present promising alternatives to nerve autografts [55]. Zhang et al. explored the potential of gingiva-derived mesenchymal stem cells, cultured in a 3D-bioprinted hydrogel, to be converted into Schwann cell precursor-like cells and incorporated into natural NGCs [56]. These functionalized NGCs significantly improved axonal regeneration and functional recovery in a segmental facial nerve defect model in rats [56]. While in vivo studies are promising, clinical trials are necessary to validate the results and overcome regulatory hurdles for the translation of additively manufactured nerve grafts.

### 3.5. Other Soft Tissue Reconstruction Considerations

For soft tissue bioprinting therapies to be successfully implemented in the clinical space, they must achieve sustained and sufficient vascularization post-implantation [57]. However, traditional syringe-based bioprinting techniques have struggled to consistently produce perfusable constructs with hierarchical branching at microvascular resolutions [22]. Hooper et al. attempted to address this drawback by introducing a novel bioprinting device that allows for the mid-extrusion control of material and channel properties, effectively producing wide cell-laden hydrogel sheets capable of producing branching channels encompassing the resolution range of microvascular vessels [22]. The customizable hydrogel sheets maintained over 90% cell viability in vitro [22]. Furthermore, Cheng et al. 3D-bioprinted a gelatin methacrylate hydrogel, seeded with adipose-derived mesenchymal stem cells (ADSCs) combined with human umbilical vein endothelial cells, to overcome the limitation of oxygen and nutrient diffusion in large area defects [58]. This study demonstrated improved cell viability and vascularization potential in an in vivo model [58]. Similar techniques aimed at promoting vascularization within soft tissue defects have been successfully demonstrated in other studies [59].

### 3.6. State of the Art in Plastic and Reconstructive Surgery

Three-dimensional bioprinting within the field of plastic and reconstructive surgery has evolved significantly following decades of development, demonstrating strong therapeutic potential in each of the tissue models discussed above. However, few 3D-bioprinted products, including human skin, have reached clinical trials. In breast reconstruction, the current standard of care involves either an autologous tissue flap or implant-based treatment. However, there are significant complications associated with flap failure and implant infection rates [60]. Three-dimensional bioprinting demonstrates the potential to overcome these shortcomings by employing cell-laden lipo-transfer scaffolds, which may be of either biological or synthetic origin. In the preclinical studies demonstrated above, encouraging results were noted. Collagen-based scaffolds have shown both the ability to produce significant amounts of adipose tissue as well as the generation of growth factors necessary for adipogenesis and angiogenesis [61]. However, one of the major challenges to their application is the uncontrolled degradation and robust immune response observed in vivo [62].

Synthetic scaffolds have the potential to overcome these challenges; however, their major drawback includes lower native cellular affinity to the synthetic materials. It has been hypothesized that a hybrid construct of a polymer core with biologic matrices may overcome these disadvantages; however, this approach has met with limited success [63]. In plastic and reconstructive surgery, burn tissue 3D bioprinting may be the closest to reaching clinical applications. In the preclinical models of burn wounds discussed, it is clear that biomaterial constructs complemented by stem cell-based tissue engineering techniques yield encouraging results as full-thickness defects have shown adequate regeneration [64]. However, a common limiting factor in these techniques is adequate vascularization after transplantation, which may be due to the current resolution limitations of 3D bioprinters [65]. As 3D bioprinting technology continues to advance, and techniques in employing these technologies are refined, the clinical translation of these models in plastic and reconstructive surgery will be realized.

## 4. Bioprinting in Orthopedic Surgery

In addition to plastic and reconstructive surgery, the use of 3D bioprinting has also become prevalent in orthopedic surgery. An abundance of resources has been allocated to developing implantable biomaterials that help regenerate bone and soft tissue (cartilage, tendons, ligaments) that have been historically difficult to heal. The pathway from the laboratory (bench-top) to the clinic (bedside) remains the same. First, in vitro studies develop new materials, printing modalities, and designs that are theorized to have adequate mechanical properties and biological stimulation to allow for proper native tissue regeneration. Often, these materials use a combination of 3D printing and a cell line (i.e., MSCs, chondrocytes, pre-osteoblasts, etc.) to create a bioactive scaffold/construct. Next, in vivo, preclinical studies translate these cellular studies into animal models to determine the osteogenic, chondrogenic, or tenogenic properties in living species. It is the combination of in vitro development and in vivo testing that deems whether novel implantable designs are suitable for human trials. This section will discuss these 3D-bioprinted designs with respect to orthopedic surgery applications.

### 4.1. Bone Tissue Reconstruction

The three-dimensional printing of grafts to restore bone tissue has been studied for decades in hopes of designing regenerative implants with the capacity to heal segmental bone defects or fractures [66,67,68,69,70,71,72,73,74,75]. Various bioprinting techniques have been investigated to fabricate scaffolds with bioactive properties that allow for osteogenesis while maintaining mechanical stability. Studies on these techniques typically analyze a few properties of the 3D printing process to determine superiority to controls, or previously utilized techniques (Table 2). First, the bioinks are subjected to rheological analysis, studying ink viscosity and printability. Next the scaffolds, laden with various cell types, are tested for cell viability, proliferation, and cytotoxicity to ensure biological functionality. The scaffolds are also frequently tested for mechanical stability, porosity, swellability, and imaged via microcomputed tomography (micro-CT) or electron microscopy. Further, in vitro studies often test for proper cell differentiation, gene expression analysis, and osteogenesis.

In recent literature, there have been numerous innovations for 3D printing bone scaffolds in vitro, with twenty-nine papers included in this review of 3D printing for bone regeneration. Of these, twenty-six utilized an extrusion-based bioprinting technique and three utilized stereolithography. Additionally, a variety (i.e., autoclave, ethanol, and ethylene oxide) of sterilization techniques were utilized; however, UV sterilization was the most common. The novelty of the studies often stemmed from altering the bioink or changing cell types used for scaffold preparation to promote osteogenesis. Cells used in these studies consisted of MSCs (bone marrow or adipose-derived), pre-osteoblast cells, osteoblasts, HUVECs, osteosarcoma cells, and dermal microvascular endothelial cells. Cells require different conditions to stimulate osteogenic differentiation and/or osteogenesis. The included studies had culture times ranging from 2 to 56 days, with the most common culture time being 14 days [95,96]. Amler et al. specifically studied the osteogenic properties of scaffolds seeded with mesenchymal progenitor cells (MPCs) from different anatomic locations, namely alveola, iliac crest, fibula, and mastoid bone [97]. They found that periosteal-derived mastoid MPCs demonstrated the highest bone mineralization, gene expression, and osteogenic differentiation relative to other biopsy sites [97]. Bari et al. studied MSC secretome, which is a noncellular formulation of various proteins and extracellular vesicles released from MSCs [95]. They found that this treatment, at various delivery rates, displayed excellent osteoinductivity with accelerated bone formation [95].

#### 4.1.1. Bioink and Additive Selection

The majority of studies included in this review attempted to modify and analyze various bioink properties (i.e., printability and regenerative capacities) that were utilized in 3D printing. The most commonly utilized bioinks were combinations of gelatin and alginate hydrogels. Gelatin is a product of collagen that demonstrates high cell viability at low concentrations and improved stiffness at high concentrations [98]. Leucht et al. and Stolarov et al. both studied the utility of various derivatives of gelatin, creating an outline for how their ratios affect mechanical stability, cell viability, and print fidelity [98,99]. Alginate is another natural polysaccharide commonly used as a hydrogel base that has good rheological properties and is highly modifiable [100]. Gonzalez-Fernandez et al. demonstrated that the modifications of alginate have an effect not only on printability but also cell compatibility and osteogenic potential [100]. They observed that alginate–CaCl_2_ exhibited increased osteogenic differentiation and cell–matrix interactions [100].

Several studies analyzed the addition of polymers to improve printability, lower costs, or increase bioactivity [83,101,102]. Liu et al. successfully created bone scaffold adding oligo (poly(ethylene glycol) fumarate) (OPF) to gelatin [83]. Chrungoo et al. found that adding polyelectrolyte complex aggregates of gelatin and chitosan improved cell adhesion and the osteogenic activity of the scaffold [83].

Five of the included studies analyzed the addition of naturally occurring components to bioink, including ECM, fibrinogen, collagen, cellulose, and egg white [103,104,105,106,107]. All of the aforementioned compounds provided improved osteogenesis. Guo et al.’s addition of collagen demonstrated improved mechanical strength relative to scaffolds without collagen [105]. Im et al. also discovered improved mechanical properties with their addition of tempo-oxidized cellulose nanofibrils (TOCNFs) and polydopamine nanoparticles (PDANPs) [106]. TOCNFs and PDANPs have strong binding affinity to calcium, imparting mechanical strength, while PDANPs have also demonstrated osteoinductive properties [108,109].

Three included studies analyzed the addition of bioactive glass (BAG) to bioink [110,111,112]. BAG has been shown to promote mineralization and induce osteogenic differentiation [113,114]. Zhu et al. and Pant et al. found the addition of BAG to be advantageous, improving mechanical stability, cell proliferation, osteogenesis, and angiogenesis [111,112]. However, Ojansivu et al. concluded that the increased viscosity of bioactive glass added to a gelatin–alginate hydrogel contributed to osteoblast-like cell death and diminished cell viability [110].

Graphene oxide, a compound known for its excellent electroconductivity, biocompatibility, and mechanical rigidity, was studied in three of the included studies [79,115,116]. Lafuente-Merchan et al. discovered that the addition of graphene oxide to alginate improved the rheological properties of the bioink and demonstrated a greater gene expression of osteogenic markers [79]. Choe et al. confirmed these results and discussed additional mechanical stability in scaffolds composed of graphene oxide [115]. Four included studies analyzed the addition of inorganic compounds for scaffold efficacy, namely amorphous calcium magnesium phosphate (ACMP), magnesium hydroxide nanoparticles, strontium-substituted hydroxyapatite nanoparticles, and octacalcium phosphate [117,118,119,120]. Kim et al. determined that this inorganic addition improved biocompatibility and osteogenesis; however, excess magnesium concentration showed an inhibitory effect on cell proliferation and mineralization [120]. Work conducted by Alcala-Orozco et al. was in agreement, demonstrating that scaffolds containing magnesium hydroxide nanoparticles had enhanced bone matrix deposition [117]. Additionally, Fischetti et al. went on to determine that inorganic nanoparticles can be added to a bioink up to 1% w/v concentration while still maintaining printability and biocompatibility [119]. Conversely, Anada et al. studied bioinks with octacalcium phosphate due to its affinity to convert to hydroxyapatite at physiologic conditions [118,121]. They also used a complex scaffold design with a cortical shell and central ring to resembling the bone cortex and marrow, respectively [118]. This design with octacalcium phosphate and its unique mechanical properties stimulated increased osteoblast generation and angiogenesis via capillary network formation [118]. Another study by Chiesa et al. found success in vascularizing bone tissue by seeding their scaffold with MSCs for osteogenic differentiation, followed by sequential seeding with HUVECs for angiogenic differentiation. This method allowed for tissue that better resembled native bone [122].

Aside from adding various materials to bioink, other studies focused on how the mechanical property of the scaffold affects bone regeneration. Zhou et al. concluded that porous polylactic acid (PLA) scaffolds were superior to solid PLA scaffolds, resembling native bone and exhibiting better cell attachment and mineralization [123]. Interestingly, Zhang et al. discovered that when using a hydrogel composed of alginate and gelatin, softer scaffolds outperformed harder scaffolds in terms of DNA content, osteogenic differentiation, and mineralization [124]. Softer scaffolds are composed of lower alginate content relative to gelatin. Two included articles also focused on the post-processing mechanical loading of scaffolds. Osteoblasts are known to have biological responses to mechanical compression and shear stress that aid in bone remodeling [125]. Zhang et al. demonstrated that immediate mechanical compressive loading on day 1 of MSC seeding resulted in increased organoid stiffness, density, and osteoblast differentiation [96]. Similarly, Mainardi et al. found that stimulating scaffolds with shear stress induced a higher bone volume and increased mineralization [82].

All the in vitro studies provided the groundwork for animal models for bone regeneration via 3D printing in vivo. Thirteen in vivo papers were included in this review of 3D printing for bone regeneration. Of these papers, the most common 3D printing technique was extrusion-based bioprinting (n = 7), followed by stereolithography (SLA) (n = 3), fused deposition modeling (FDM) (n = 1), digital light processing (DLP) (n = 1), and mechanical dispense (n = 1). Cells used in these studies consisted of embryonic fibroblasts, osteoblast-like cells, bone marrow MSCs, endothelial progenitors, HUVECs, umbilical cord MSCs, and articular cartilage progenitors. The included studies had culture times ranging from 7 days to 35 days, with the most frequent culture time being 21 days [85,93]. The time in vivo ranged from 2 weeks to 12 weeks [63,93].

Several studies analyzed the development of bone regenerative scaffolds using novel bioinks. Nulty et al. used a fibrin-based hydrogel with gelatin methacrylate and nano hydroxyapatite-coated PCL in a critically sized defect model of rabbit femurs [93]. They found that revascularized tissues lead to accelerated angiogenesis with excellent bone regeneration [93]. Conversely, Kumari et al. studied a rat model with scaffolds composed of methacrylate-Κ-Carrageenan hydrogel with silica nanoparticles [126]. Κ-Carrageenan is a naturally occurring polysaccharide expressed in the ECM of human bone with excellent mechanical properties and confirmed in vitro success [127]. This rat model demonstrated confirmed printability and osteogenic properties [126]. Shi et al. studied a scaffold composed of hydroxyapatite, aragonite, and gelatin in a rat tibia critically sized defect model [128]. The scaffold displayed fast biodegradability and accelerated bone formation, which was attributed to the biodegradability of aragonite [128,129]. Lastly, Zhang et al. used an anisotropic bicellular (articular cartilage progenitor cells and bone marrow MSCs) hydrogel to create an osteochondral scaffold in rabbit femoral grooves [88]. The scaffold demonstrated a biomimetic osteochondral interface with differentiated chondrocytes and osteoblasts [88].

**Figure 4:**
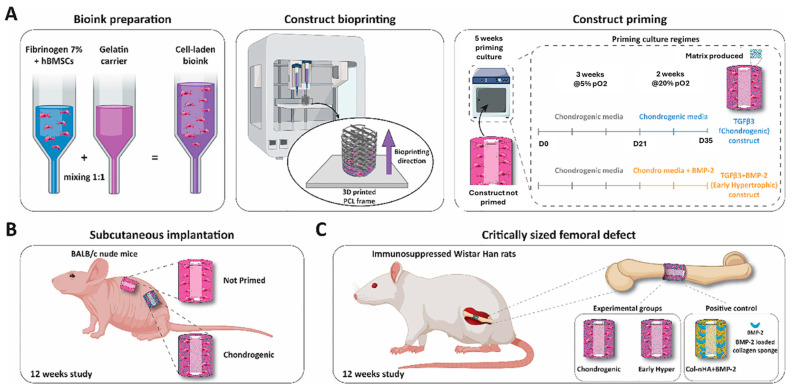
A schematic illustration of bioprinting cartilaginous templates for orthopedic applications highlighting (**A**) 3D-bioprinted PCL reinforced cartilaginous template fabrication, and schematic of (**B**) subcutaneous implantation; and (**C**) implantation into critically sized femoral defects. Adapted from Pitacco et al. [85] under the terms of the Creative Commons Attribution—Noncommercial 4.0 License. https://creativecommons.org/licenses/by/4.0/ (accessed on 11 July 2024).

Four included studies analyzed the effect of adding a biologic molecule to the scaffold [63,85,90,130]. Sun et al. showed in a mouse femoral defect model that BMP-4 improved angiogenesis and osteogenesis [90]. Abu Awwad et al. demonstrated that runt-related transcription factor (Runx2)-loaded microparticles improved osteogenesis in their mouse femoral model [63]. In another femoral model, Lauer et al. described the effect of stromal-derived factor 1 (SDF-1) on osteogenesis, where SDF-1 induced more regeneration than scaffolds with BMP-7 or negative controls [130]. Lastly, in a rat femoral condyle model, Pitacco et al. displayed that BMP-2-loaded collagen sponges improved mineralization and vascularization, producing a viable osteogenesis and chondrogenesis model for the osteochondral interface (Figure 4) [85]. However, they did find that this was associated with greater heterotopic bone formation. These studies provide clear examples of how biologic molecules can aid in 3D-printed tissue regeneration but only scratch the surface of the abundance of biologics being studied.

#### 4.1.2. Scaffold Drug Delivery and Antimicrobials

Drug delivery is another aspect of biologic administration that must be considered in tissue regeneration. Kondiah et al. developed a scaffold, optimized by artificial neural networks, which was loaded with simvastatin and implanted into a human clavicle [91]. This scaffold exhibited sustained drug release over 20 days and successfully resulted in bone regeneration [91]. This artificial neural network technology can be further used to optimize delivery for various drugs such as antibiotics or other growth factors.

Two of the included studies analyzed the antimicrobial properties of scaffolds in an effort to eliminate infectious complications. Shokouhimehr et al. utilized implants with superparamagnetic iron oxide nanoparticles (SPIONs) in a rat femoral model [86]. SPIONs are known to be great antibacterials, even killing antibiotic-resistant bacteria [131]. The model found that these scaffolds had enhanced bacteriostatic properties and achieved complete bone bridging [86]. Conversely, Wang et al. loaded their PCL-BAG scaffolds with doxycycline, which successfully prevented infection and mediated the release of BMP-2, resulting in bone regeneration [132].

#### 4.1.3. Higher Complexity 3D Printing Designs

Two other studies used complex 3D printing designs to create scaffolds that better resembled native tissue. In Sun et al.’s mice femur model, the authors designed and printed a complex scaffold, complete with medullary canals, Haversian canals, and Volkmann canals [90]. Upon healing, the regenerated bone had adequate bridging and mimicked the native cell pattern [90]. Lastly, Chae et al. created a spatially graded tendon-bone scaffold using tendon-derived decellularized ECM, bone decellularized ECM, and bone marrow MSCs [133]. They implanted these scaffolds in rat shoulders, exhibiting healing and adequate biomechanical strength [133]. These studies display how improved 3D printing design and precision can lead to more complex scaffolds and ultimately further bone regeneration.

### 4.2. Cartilage Reconstruction

The 3D printing of scaffolds for cartilage repair requires alternative considerations compared to bone. First, cartilage is natively an avascular tissue, posing a challenge for deliverance of cellular nutrients [134]. Second, cartilage is a soft tissue, requiring different printing modalities and materials to develop a biomimetic construct [135]. Lastly, cartilage often attaches to bone, adding another layer of complexity to develop constructs with adequate functionality and adhesion to natural bone [134]. The studies developing 3D-printed cartilage analyzed largely the same variables as for 3D-printed bone (Table 2). The variables differed from bone regeneration by using different cellular markers to measure chondrogenesis and gene expression analysis (glycosaminoglycans (GAG), collagen type II (COLII), etc.).

Twenty-two in vitro papers discussing 3D printing cartilage were included in this review. Additionally, there were three papers discussing 3D printing tendon and one paper discussing 3D printing muscle. Of these, extrusion-based printing was most frequently utilized (n = 16), followed by inkjet-based printing (n = 3), stereolithography (n = 2), fused deposition modeling (n = 2), filamented light (n = 1), and mechanical dispense (n = 1). A variety of sterilization techniques were used, including autoclave, ethylene oxide, UV irradiation, gamma irradiation, and ethanol. Once again, each study differed by the type of cell used for proliferation or by modifying the bioink. Cells used in these studies consisted of animal or human chondrocytes, osteoblasts, MSCs, bone marrow MSCs, and chondrogenic cells. One study by Laternser et al. used human skeletal muscle cells and rat tenocytes in a gelatin-based hydrogel to 3D-print a connected muscle–tendon construct with retained functionality [136]. The included studies had culture times ranging from 3 days to 56 days, with the most frequent culture time being 21 days [137,138].

Four of the included studies used novel 3D printing techniques to create viable soft tissue constructs. Baena et al. used a fused deposition modeling (FDM) volume-by-volume method, which was a sequential process of layering followed by hydrogel filling [139]. This method was proven to improve chondrocyte survival by avoiding elevated temperatures associated with other 3D printing techniques [139]. Van Loo et al. used a single-step “in-air microfluidic” biofabrication of spheroid forming hydrogels [140]. This process avoided excessive cleaning steps and improved the process speed, thereby improving overall efficiency and clinical translatability. Finally, Galarraga et al. used a fused deposition modeling technique with a norbornene-modified hyaluronic acid macromer bioink [137]. This process minimized shear stress during printing and eliminated the need for rheological additives, increasing cell viability [137].

#### Bioink and Additive Selection

The majority of the studies commonly modified the specific bioink used in 3D printing, analyzing its printability and regenerative properties. As with bone regeneration, the most common bioinks used were combinations of gelatin and alginate hydrogels. Lam et al. developed several derivatives of gelatin-based cartilage constructs and found that gelatin methacrylate hydrogel demonstrated a higher gene expression of collagen relative to constructs made from hyaluronic acid methacrylate [141]. Conversely, Gorroñogoitia et al. studied the properties of various alginate-based hydrogels in cartilage construction [142]. They determined that high molecular weight alginates demonstrated superior printability. However, low-molecular-weight alginate hydrogels with guluronic acid demonstrated superior mechanical stability [142,143]. Barcelò et al. confirmed the adequate chondrocyte differentiation of alginate-based hydrogels but also added that partially oxidized alginates enabled superior degradation rate control [144].

Many studies also discussed the addition of various novel compounds to bioink. Several studies added naturally occurring compounds to hydrogel-based bioinks to better stimulate chondrocyte differentiation and proliferation. Behan et al. added methacrylated ECM to a gelatin-based hydrogel [80]. They found that the rheological properties of the bioink were dependent on the ECM concentration; however, all concentrations demonstrated high cell viability and chondrogenesis [80]. Toprakhisa et al. also found success in developing an ECM-based tendon construct from bovine tendons, which showed promising biomimetic properties and the maintenance of the tendon microarchitecture [138]. Rathan et al. developed a cartilage construct, adding an ECM to an alginate-based hydrogel [84]. The addition of the ECM enhanced chondrogenesis and adding PCL improved mechanical properties, further resembling native cartilage [84]. Similarly, Lafuente-Merchan et al. added nanocellulose composed of ECM components, namely chondroitin sulfate (CS) or dermatan sulfate (DS), to an alginate-based hydrogel [76]. CS and DS are both glycosaminoglycans naturally found in articular cartilage, known for their mechanical strength and cell differentiation stimulation, respectively [145,146]. They concluded that the bioink composed of CS had improved rheological and mechanical properties; however, the DS bioink displayed improved metabolic activity with increased gene expression activity [76]. Muller et al. also supplemented an alginate-based hydrogel with nanocellulose, which was found to demonstrate improved cell proliferation and collagen synthesis [147]. They also found a relationship between biological performance and nozzle geometry, with smaller diameter nozzles compromising chondrocyte proliferation [147]. Rather than just using an ECM or components of the ECM, Kiyotake et al. created a hydrogel from complete devitalized cartilage [77]. This product demonstrated paste-like rheological characteristics and fast crosslinking, which may be useful in the clinical setting [77].

Three included studies also discussed using silk in cartilage constructs [148,149,150]. Chakraborty et al. studied the addition of silk fibroin to gelatin-based hydrogels wherein silk fibroin improved chondrogenesis and decreased hypertrophic markers, further resembling native cartilage [149]. Conversely, Bandyopadhyay et al. and Ni et al. used silk-based bioinks without gelatin or alginate [148,150]. Bandyopadhyay et al. used a silk methacrylate-PEG diacrylate hydrogel that demonstrated adequate printability with an ideal porous structure that is suitable for cartilage regeneration [148]. Ni et al. utilized a double network system composed of silk fibroin hydroxypropyl methyl cellulose. The double network system allowed for improved mechanical properties by combining two types of polymers while maintaining excellent biocompatibility [150].

Other polymers have been added to hydrogels in hopes of finding optimal cartilage regeneration. Olmos-Juste et al. created a construct using an alginate-based hydrogel with waterborne polyurethane due to its non-cytotoxic nature and stable mechanical properties [81]. They demonstrated maintained structural integrity and adequate cell viability [81].

Two studies analyzed the effects of adding inorganic molecules to bioinks. Kosik-Koziol et al. attempted to develop a calcified cartilage construct by adding β-tricalcium phosphate to alginate or gelatin methacrylamide hydrogels [151]. With both hydrogels, they successful created calcified osteochondral tissue with cartilaginous hypertrophy [151]. This tissue has potential for articular cartilage application due to the calcification that must occur at the calcified cartilage zone of the bone–cartilage interface. Aside from cartilage, Li et al. studied the addition of calcium chloride and PCL to alginate hydrogel for a tendon construct that improved cell alignment [152].

Finally, three studies analyzed the addition of biologic molecules in cartilage regeneration [153,154,155]. Zhu et al. and Henrionnet et al. both studied the effect of TGF-β1 on cartilage regeneration, concluding that the biological molecule improved chondrogenic differentiation [153,155]. Further, Henrionnet et al. found that TGF-β1 combined with BMP-2 provided maximal ECM synthesis, exhibited by GAG and COLII expression [153]. Conversely, Majumder et al. identified that the administration of Interleukin-1 receptor receptor antagonist (IL1Ra) enhanced articular cartilage regeneration while inhibiting hypertrophic and inflammatory markers [154]. The presence of Interleukin-1 (IL1) has been shown to contribute to cartilage degeneration, and thus, its inhibition ensures maximal regeneration [156].

The in vitro studies provided the groundwork for in vivo animal models for soft tissue regeneration via 3D printing. Ten in vivo papers were included in this review of 3D printing for soft tissue regeneration. Of these, extrusion-based bioprinting was the most common technique (n = 7), followed by stereolithography (n = 3). Cells used in these studies consisted of synovium-derived MSCs, HUVECs, bone marrow MSCs, adipose-derived MSCs, and articular chondrocytes. The included studies had culture times ranging from 5 days to 42 days, with the most frequent culture time being 21 days [157,158]. The time in vivo ranged from 1 week to 24 weeks [159,160].

**Figure 5:**
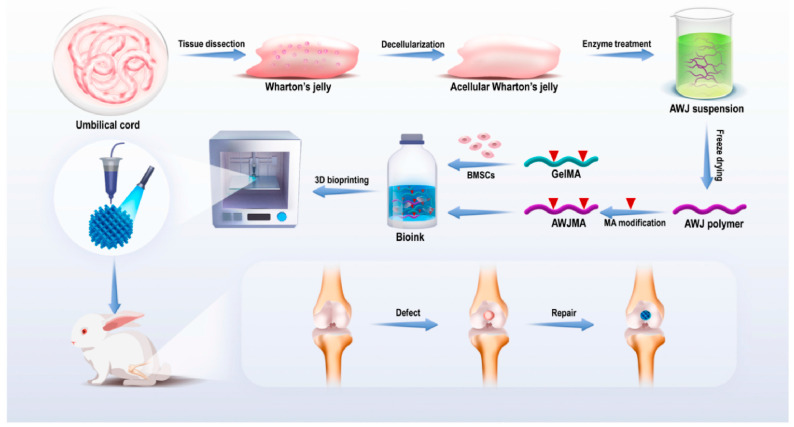
Schematic illustration of full-thickness articular cartilage defect repair using 3D-bioprinted cartilage-mimicking substitutes based on photo-crosslinkable Wharton’s jelly bioinks, as demonstrated by Hu at al. [92], reprinted under the terms of the Creative Commons Attribution—Noncommercial 4.0 License. https://creativecommons.org/licenses/by-nc-nd/4.0/ (accessed on 11 July 2024) Abbreviations—AWJMA: methacryloyl-modified acellular Wharton’s jelly; AWJ: acellular Wharton’s jelly; MA: methacrylation.

Several studies analyzed the development of cartilage regenerative constructs using novel bioinks. Nulty et al. utilized a fibrin–gelatin–hyaluronic hydrogel construct that was pre-vascularized with HUVECs and cultured with bone marrow MSCs in a rat knee cartilage model [89]. After 4 and 8 weeks, all cartilage exhibited successful mineralization, but pre-vascularized constructs performed better [89]. In another knee cartilage model, Hu et al. developed a cartilage-mimicking construct from an acellular Wharton’s jelly-gelatin methacrylate hydrogel [92]. The cartilage demonstrated promising biocompatibility with suitability physicochemical properties (Figure 5) [92]. Sun et al. created a separate composite construct composed of PCL with gelatin, fibrinogen, HA, and glycerol [158]. It was printed in a gradient structure, which exhibited greater cartilage repair in a rabbit knee model relative to non-gradient structures [158]. Others used alternative hydrogel materials to create cartilage constructs. Zhang et al. used silk fibroin with a decellularized ECM in mice and this demonstrated high mechanical strength and a suitable degradation rate [161]. Conversely, Huang et al. used a novel naringin-based scaffold in a rabbit knee model [157]. Naringin is a compound naturally found in plants that has been proven to facilitate chondrogenesis and increase COLII and GAG expression [162]. They proved that naringin alone had similar bioactivity to mathylacryloyl-modified naringin, both of which facilitated cartilage repair [157].

Four of the studies analyzed the effect of adding a biologic molecule to the cartilage construct, examining TGF-β1, TGF-β3 BMP-4, and growth differentiation factor 5 [87,159,160,163]. All these biologic molecules contributed to chondrogenesis and the success of cartilage regeneration. Of note, the combined TGF-β3 and BMP-4 treatment also was seen to induce micro vessel ingrowth [163]. These studies acknowledge the importance of biologic administration for the successful regeneration of cartilage via 3D printing.

Finally, one study tested the effects of mechanical stimulation on tendon and ligament regeneration in a mouse model. Chae et al. developed tendon and ligament constructs out of a decellularized ECM with gelatin and PCL [164]. The constructs were preconditioned by being placed under static tension, which has been proven to induce cell differentiation [165]. The study found that preconditioned constructs resulted in functional tissue with a superior regenerative capacity [164].

### 4.3. State of the Art in Orthopedics

Three-dimensional printing in orthopedics is a field with great promise, despite its minimal presence in the clinic. Clinical orthopedic 3D printing is being used most frequently for patient education and surgical planning [166,167]. Models are printed to explain complex anatomy to patients pre-operatively while various simulations and surgical guides can be printed for surgeon training and operative planning [168]. To date, these 3D printing applications have been shown to improve patient satisfaction [167,169]. Despite these effective 3D-printed devices, there has been a minimal use of patient-specific implantable devices in the clinic for several reasons. First, 3D printing is not readily accessible to all institutions but is commonly limited to large academic centers [170]. Additionally, the 3D printing process can have long processing times and increased costs, making it less appealing [171]. Moreover, implantable devices are highly regulated, often taking many years to translate preclinical results to clinical applications [170]. However, as 3D printing becomes more available and efficient, costs and processing times continue to decrease, making this a viable option in the years to come [166]. Three-dimensional printing in orthopedics provides an opportunity for extremely accurate patient-specific devices that will decrease operative times and may decrease complication rates. Additionally, the new materials being developed, as described above, show potential for improved healing and tissue regeneration, which may lead to better outcomes for patients. Overall, the 3D printing processes provide an advantageous orthopedic treatment modality and will likely become more available following further clinical trials and regulatory approval.

## 5. Bioprinting in Ophthalmology

Three-dimensional bioprinting has gained significant research interest in the field of ophthalmology [172]. Bioprinting in ophthalmology represents a frontier in medical technology, offering new prospects for treating a range of eye conditions. This innovative approach leverages 3D printing technologies to create complex, cell-laden structures that mimic various eye components, from corneas to retinas. This technique not only helps restore vision but also serves as a valuable tool in drug testing and disease modeling and may provide a more accurate representation of human disease processes.

### 5.1. Corneal Reconstruction

The cornea is a transparent, dome-shaped structure at the front of the eye that covers the iris, pupil, and anterior chamber. It is the eye’s primary light-focusing structure, being responsible for approximately 65–75% of the optical refractive power. It comprises five distinct layers: the outermost epithelium, Bowman’s membrane, the stroma, Descemet’s membrane, and the innermost endothelium [173]. Each layer has unique structural and functional properties that must be accurately replicated in a 3D-bioprinted construct. Biomechanically, the cornea serves as an air–liquid interface on the eye’s surface, enduring the pressures from the aqueous humor and the tension of extraocular muscles [174]. As an anisotropic soft tissue rich in water, the cornea displays a unique stress response, exhibiting properties of both solids and viscous liquids [174]. The surgical applications for corneal bioprinted structures have each been examined, including their uses in full-thickness keratoplasty and partial-thickness lamellar keratoplasty.

While full-thickness corneal transplants comprise a future goal of corneal 3D bioprinting, the main challenges for translation of this treatment modality in vivo include the development of a graft with proper tensile strength, an appropriate curvature, biocompatibility considerations, and a maintenance of optical clarity. A full-thickness model replicating these complex biomechanical properties has yet to be developed [175]. Critical challenges in corneal bioprinting involve ensuring the bioprinted cornea exhibits long-term survival, biomechanical stability, light transmission, and effective cell-to-cell interactions [176,177].

Numerous studies have been dedicated to regenerating the stromal layer of the cornea, a critical area of research due to the stroma’s vital role in maintaining clear vision (Table 3). The corneal stroma, making up the bulk of the cornea’s structure, is essential for its transparency and refractive properties. Consequently, advancements in stromal regeneration could lead to breakthrough therapies that restore vision or prevent vision loss in patients with corneal disorders. A significant challenge in the printed corneal graft’s structural integrity is the integration of the stromal layer, which makes up approximately 90% of the corneal thickness [178]. Campos et al. developed a functional corneal stromal equivalent using a collagen-based bioink and primary human corneal stromal keratocytes, with the printed constructs demonstrating appropriate optical and structural properties [176]. Bektas et al. conducted a study to develop 3D-bioprinted corneal stroma substitutes using GelMA hydrogels [177]. The hydrogels exhibited collagen types I and V, maintaining transparency comparable to the native cornea [177]. This study demonstrated that GelMA hydrogel scaffolds can be a viable substitute for the cornea stroma. Building on this research, subsequent studies were initiated to assess the efficacy of GelMA hydrogels in bioprinting corneal stromal cells and to compare the performance of two different concentrations of the bioink. This study tested GelMA with corneal stromal cells as a bioink in concentrations of 7.5% and 12.5% for bioprinting the corneal stroma [179]. The 12.5% GelMA scaffolds more effectively replicated the transparency and water content of native corneal stroma and better supported corneal stromal cell functions than the 7.5% GelMA scaffolds, establishing this as a superior bioink for corneal applications [179]. Later studies broadened the scope to include fiber-reinforced GelMA hydrogels and chemical factors [180]. These investigations aimed to enhance the differentiation of limbal stromal stem cells into keratocytes or fibroblasts for corneal stroma regeneration. They examined the impact of various topologies (3D fiber hydrogel, 3D GelMA hydrogel, and 2D culture dish) and chemical stimuli (serum, ascorbic acid, insulin, and β-fibroblast growth factor (β -FGF)) to determine optimal conditions for this model. The results suggest that the fiber hydrogel, when used with serum-free media containing insulin, β-FGF, and ascorbic acid, creates an optimal environment for maintaining the keratocyte phenotype and regenerating the damaged corneal stroma [180]. In addition, the cornea stroma has been developed using a multi-material strategy with using both a stiff acellular bioink as well as a soft cell-laden bioink, allowing for the mimicking of the heterogeneous structure of the corneal stroma [181].

Advancements in bioprinting systems and bioink materials have allowed for enhanced graft integrity and biocompatibility. Various bioprinting techniques, including extrusion-based printing, digital light processing (DLP), and electromagnetic micro-valve bioprinting, have been explored to fabricate corneal constructs [182,188,189]. The suitability of bioink for corneal bioprinting is determined by its rheological properties and ability to crosslink [190]. When selecting a bioprinting method, it is crucial to consider the target tissue and the primary objective of the bioprinted construct. Essential criteria for selecting corneal bioink include the resolution, cell viability, and biomaterials used in printing [191]. Thus, carefully selecting bioinks based on their properties and the bioprinting method suited to specific clinical needs is pivotal in ensuring the successful fabrication and implementation of functional corneal constructs.

**Figure 6:**
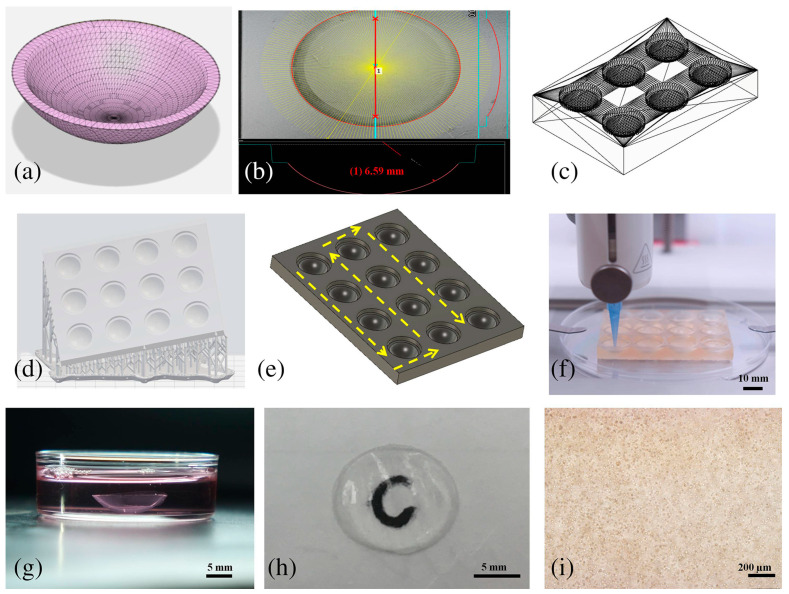
Human corneal stroma and base support scaffold apparatus development showing (**a**) the average corneal stroma dimensions modeled in CAD; (**b**) bright-field image of single well of support scaffold; (**c**) 3D model of 6-well corneal scaffold; (**d**–**f**) support and raft preparation for SLA printing, printing path, and high-throughput printing; (**g**) 3D-bioprinted cornea placed within media after the crosslinking process; (**h**) pictograph of cornea depicting its transparency through the letter “C” in the background; and (**i**) bright-field image of 3D-printed cornea using inverted microscope. Reprinted with permission from the work by Kutlehria et al. [182] under license number 5824890412725. Copyright 2020 John Wiley and Sons.

Building on these foundational insights, advancements in bioprinting techniques have significantly enhanced corneal regeneration, utilizing innovative materials and technologies to mimic the structure and function of the native cornea closely. In one study, a cornea-derived decellularized extracellular matrix was employed as a bioink for corneal regeneration [192]. This bioink showed collagen and glycosaminoglycan levels similar to the native cornea and maintained essential transparency [192]. Additionally, its safety and ability to maintain keratocyte features were on par with clinical-grade collagen [192]. DLP is another high-throughput technique that has demonstrated the ability to preserve cell viability and holds promise for future full-thickness biofabrication strategies [193]. This strategy for developing a stromal equivalent was achieved using an extrusion-based technique, along with a scaffold printed on a stereolithographic printer in tandem with micro-transfer molding (Figure 6) [182]. Another study by He et al. investigated the biomimetic epithelium/stroma bilayer implant for corneal regeneration using DLP technology [194]. A bi-layer long-chain poly(ethylene glycol) diacrylate (PEGDA)-GelMA hydrogel corneal scaffold was printed, featuring aligned layers of rabbit corneal epithelial cells and rabbit adipose-derived mesenchymal stem cells within a fibrous stroma [194]. In a rabbit keratoplasty model, this scaffold effectively sealed defects, supported re-epithelialization, and promoted stromal regeneration. The scaffold’s precise microstructure and strategic cell placement optimized the corneal regeneration microenvironment [194]. Concerning bioinks, some of the main challenges include balancing the printability while maintaining the biocompatibility of the graft. Mörö et al. reported on the development of a bioink that preserved both of these features whereby the corneal structure demonstrated excellent ex vivo integration with the host tissue and in vitro nervous innervation [195]. However, future research must utilize these bioprinting techniques to replicate the optical and cellular complexity of the human cornea.

While bioprinting in the corneal stroma is relatively well established, the 3D bioprinting of the corneal endothelium offers a promising technological advancement. This technology enables the production of high-resolution scaffolds with precise geometric features, which are crucial for cell attachment, proliferation, and differentiation within corneal endothelium tissue engineering. Human corneal endothelial cells are incapable of regenerating through cell division in vivo. Corneal endothelial pathology occurs in Fuchs endothelial corneal dystrophy and can lead to corneal edema and vision impairment. Grönroos et al. demonstrated that human pluripotent stem cell-derived corneal endothelial cells can be successfully printed using a covalently crosslinked hyaluronic acid bioink [196]. The bioink, tailored for human pluripotent stem cell-derived corneal endothelial cells, effectively supported their viability and printability. STEM121-positive cells were identified on the Descemet membrane in rat and porcine corneas up to 10 days post-transplantation, confirming the bioink’s biocompatibility [196]. Seven days after bioprinting, these cells maintained viability and displayed a polygonal shape with appropriate expression of tight junction-associated protein zonula occludens-1 (ZO-1), Na+/K+ ATPase, and activated leukocyte cell adhesion molecule (CD166), although mesenchymal-like cells were also noted [196]. This method offers potential for corneal endothelial transplants and paves the way for progress in bioprinting a full-thickness human cornea.

### 5.2. Retinal Reconstruction

The retina is a complex, multi-layered tissue lining the back of the eye responsible for converting light into electrical signals to be interpreted by the brain. It contains various specialized cell types, including photoreceptors (rods and cones), bipolar cells, and ganglion cells arranged in a precise 3D architecture. Maintaining this intricate structure is crucial for light detection, image processing, and the transmission of visual information. The retina is implicated in various diseases, including diabetic retinopathy, age-related macular degeneration, and central retinal vein occlusion, which are among the leading causes of blindness both in the United States and globally [197]. The continually increasing pathology of the retina underscores the need for effective treatments, with bioprinting emerging as a potential solution. However, the complexity of the retina presents significant challenges for 3D bioprinting as recreating this layered organization with proper cell orientation and microenvironment is essential for developing functional retinal tissue equivalents.

Researchers have explored various bioprinting techniques to fabricate complex retinal constructs that mimic the native tissue structure (Table 3). The methods reported for producing retinal structures include vat polymerization, extrusion, and jetting-based bioprinting [198]. These methodologies have created retinal tissue constructs, each with variable structural and functional performances [199]. Song et al. developed a model that incorporates multiple retinal layers with the integration of an outer blood–retina barrier to study the manifestation of age-related macular degeneration [200]. The use of photocrosslinkable hydrogels, such as gelatin methacrylate and hyaluronic acid-based bioinks, to support the encapsulation and viability of retinal cells has been reported [201]. These biomaterials have been tailored to mimic the mechanical properties and microenvironment of the native retinal tissue, providing a suitable platform for cell growth and differentiation. The use of a scaffold-free 3D printing approach has been shown promise as a technique for maintaining the structural properties of the retinal layers.

Recent advances in retinal 3D bioprinting have shown promising developments for creating functional retinal tissues, which are crucial for studying disease phenotypes, conducting drug testing, and developing potential in vivo models. Central to these advancements is the focus on the retinal pigment epithelium (RPE), a single layer of polygonal cells positioned between the photoreceptor cells and Bruch’s membrane in the outermost layer of the retina [198]. The RPE is critical as it establishes the outer blood–retina barrier, regulating the flow of substances into and out of the retina, and is associated with diseases such as retinitis pigmentosa and both wet and dry forms of age-related macular degeneration (AMD) and Stargardt’s disease [202]. Various bioinks have been studied for their ability to preserve retinal function when exploring solutions. Notably, Bruch’s membrane-derived extracellular matrix has shown enhanced functionality in the RPE compared to conventional laminin bioinks [186].

Similarly, retinal tissue-derived bioinks have proven conducive to 3D retinal cell printing, facilitating cell differentiation compared to collagen-based bioinks [184]. Masaeli et al. have advanced this field by utilizing high-resolution inkjet bioprinting to produce RPE layers that maintain structural integrity and demonstrate vital immunostaining properties shortly after bioprinting [185]. Furthermore, their work revealed that significant amounts of human vascular endothelial growth factors (hVEGF) were released from the bioprinted RPE layer, confirming the formation of a functional RPE monolayer post-bioprinting [185]. This carrier-free bioprinting technique underscored the feasibility of creating an in vitro retina model with the correct layered structure and functionality for studying retinal pathology and led to further innovations. For instance, the discovery that a Bruch’s membrane-mimetic substrate (BMS) could significantly enhance RPE layer development compared to an uncoated scaffold, offering both increased levels of ZO-1 and phototransduction enzymes as well as higher transepithelial electrical resistance (TEER) for improved barrier function [185]. This BMS RPE also shows promise for in vivo transplantation as the layers can be easily removed from the scaffold for subretinal and subcutaneous transplantation. Despite these advancements, further research is necessary to develop a composite retinal graft that preserves function for applications in degenerative diseases of the retina, indicating an exciting path forward in bioprinting technologies.

### 5.3. Lacrimal Gland Reconstruction

The lacrimal gland is a vital organ in the eye socket that produces tears to lubricate the eye for comfort and clarity of vision. Without this organ, severe dry eye affects vision and quality of life. Malignancy arising from this gland is notorious for its unpredictability and universally devastating lethality, with a 10-year survival of less than 20%—virtually no patients survive beyond 15 years with the traditional treatment methods. A multi-modal treatment strategy of neoadjuvant intra-arterial chemotherapy, orbital exenteration, and radiotherapy has substantially improved 15-year disease-free survival. A new paradigm for managing the lethal lacrimal gland adenoid cystic carcinoma is the globe-sparing approach with neoadjuvant intra-arterial chemotherapy as the core element of therapy. While this approach achieves the objectives of prolonging life and preserving a seeing eye, the eye encounters dryness due to the absence of a lacrimal gland to produce tears. Investigators have developed a method to harvest the lacrimal gland progenitor cells from the patient’s remaining normal lacrimal gland to propagate a population of tear-producing lacrimal gland cells in tissue culture. These cells can then be bioprinted to create a 3D lacrimal gland organ for transplantation into the patient’s orbit that is missing a lacrimal gland. Three-dimensional bioprinting is an approach that can simultaneously print millimeter- to centimeter-sized biological constructs, including several cell types, biomolecules, and biomaterials. This technique can construct realistic 3D tissue or organ models as it can mimic the cellular arrangement of a tissue or organ while forming the 3D structure. The ability to create an artificial tear-producing lacrimal gland will be transformative and disruptive in ophthalmology.

A robotic liquid-handling EpMotion^®^ system (Eppendorf, Hamburg, Germany) is used to precisely bioprint expanded lacrimal gland progenitor cell organoids and microparticles onto biomaterial scaffolds or decellularized tissue scaffolds to bioengineer a new lacrimal gland for managing the severe post-operative dry eye condition. The robotic system can generate bioengineered glandular structures containing all the morphological features needed to restore lacrimal gland function by combining microparticle manufacturing, progenitor cell isolation, and the organoid expansion process. Expanding organoid inventory to test the assembly parameters for the bioengineered lacrimal gland before transplantation is undergoing refinement.

### 5.4. State of the Art in Ophthalmology

The advent of 3D bioprinting in ophthalmology heralds a transformative shift in treatment paradigms, offering substantial advantages over traditional approaches [203]. Unlike conventional methods that often rely on generic prosthetics or donor tissues, which can suffer from limited availability and potential immunological rejection, 3D bioprinting enables the production of patient-specific ocular tissues with enhanced precision and scalability. This method not only reduces the risks of immune rejection by incorporating autologous cells but also allows for the creation of complex, stratified structures that more accurately replicate the intricate anatomy of ocular tissues [204]. Such precision is particularly crucial in restoring the function of highly specialized tissues such as the cornea and retina, which require exacting structural and optical properties for proper vision [205]. Although significant progress has been made, 3D-bioprinted ocular tissues have not yet reached a fully functional stage for widespread clinical application. Challenges remain in ensuring long-term viability, integration into host tissues, and full optical functionality [206]. Nevertheless, ongoing research is rapidly advancing, with a focus on refining the bioprinting processes to produce tissues that meet the rigorous demands of ocular functionality [207]. The trajectory of this research is promising and suggests that fully functional, bioprinted ocular tissues may soon revolutionize therapeutic approaches in ophthalmology, offering new hope for patients with previously untreatable conditions.

## 6. Future Directions and Challenges

### 6.1. In Situ Bioprinting

Despite the comprehensive level of success of in vitro and in vivo studies, 3D bioprinting has yet to be widely applied into the clinical space. One barrier to translation is conventional 3D bioprinters’ slow response to urgent clinical needs and their requirement of high-level expertise and infrastructure [208]. For example, in traumatic injury cases, it may take multiple hours to acquire 3D imaging from the injury site and reconstruct the defect utilizing CAD software and subsequently bioprinters. The printing process itself takes a significant amount of time, requiring high level expertise and specialized facilities. By the time the implant is ready for use, a secondary surgery may be necessary, diminishing the regenerative potential of native tissue and delaying patient care [209]. Furthermore, after the construct has been bioprinted, the handling and implantation can be challenging due to potential disruption in micro- and macro-architectures (i.e., swelling or contraction) of the construct, risk of contamination during transport, and the vulnerability of neighboring native tissues [210].

In situ bioprinting (also known as in vivo bioprinting) is a rapidly developing technique aimed at combating these drawbacks. This technique represents a form of 3D bioprinting, where biomaterials/bioinks are used to print cell-laden/acellular implants directly into the defect site within the operating room [211]. By implementing this approach, the surgical team can immediately apply the treatment, preventing a delay to implantation and controlling changes that can occur to the defect microenvironment during surgical resection and debridement [209]. Furthermore, implant integration is improved as 3D geometry is more accurately matched to both the surgical defect as well as implant adhesion to surrounding tissues through in situ crosslinking [212]. In general, in situ bioprinters can be classified into one of two groups: bedside mounted printers, where the printing rate and location are controlled by CAD tools, and hand-held printers, where device printing specifications are controlled manually [213].

Various in situ bioprinting techniques have been studied for the treatment of skin wounds, bone, cartilage, and complex defects involving heterogenous tissues. Skardal et al. attempted to bioprint amniotic fluid-derived stem cells within a fibrin–collagen hydrogel directly onto full-thickness wounds in a mouse model [214]. The histological examination of their treatment group showed greater wound closure, including increased micro vessel density and capillary diameters; however, they did not compare the treatment group to ex vivo bioprinting control groups [214]. Moncal et al. attempted to address the challenge of healing critically sized calvaria defects in a rodent model using in situ bioprinting [215]. The authors intraoperatively bioprinted gene-activated matrices at calvaria defect sites, utilizing a controlled release of growth factors to enhance bone regeneration, resulting in a significantly increased bone tissue formation and coverage area compared to controls [215]. To recapitulate the complex native anatomy of heterogenous craniofacial tissues, Moncal et al. developed a hard and soft tissue composite that was implanted directly into rats in a surgical setting [216]. Their results demonstrated a ~80% skin closure and 50% bone coverage at the end of the study timeframe, highlighting bioprinting’s potential to improve clinical outcomes by the integration of multiple tissue types [216]. Overall, in situ printing is an emerging area within the field of bioprinting, representing a strong tool for intraoperative defect management with significant progress expected in the coming years [209].

### 6.2. Regulatory Oversight

New technologies in regenerative medicine, including 3D bioprinting, are under development and have the potential to disrupt many of the traditional reconstruction therapies. However, the translation of these technologies represents a significant challenge as traditional regulatory pathways (i.e., the Food and Drug Administration (FDA)) are designed for mass-manufactured therapies rather than patient-specific solutions [217]. Furthermore, the inclusion of living cells in the manufacturing process creates an added level of complexity as newly developed regulations regarding 3D printing may not necessarily be applicable to 3D-bioprinted devices [218]. Despite this lack of up-to-date regulation, the first clinical trial of a bioprinted therapy was begun in 2022, adding urgency to the need for updated guidance on clinical applications for this technology [219].

Various global regulatory bodies, including the FDA (USA), European Medicines Agency (Europe), and Therapeutic Goods Administration (Australia) are exploring modifications to their processes to better accommodate the unique aspects of 3D bioprinting [220]. These organizations generally have strict classification and standardization requirements prior to approval of a product. Classification challenges are faced by 3D-printed products as they often combine features of both medical devices and biologic products, two separate regulatory pathways [221]. Further, there is a lack of standardization in the field as various printers, biomaterials, and additives are currently under investigation [222]. Another important consideration is quality control and safety. Each bioprinting session can vary due to differences in bioink composition, cell viability, and printer calibration, creating a lack of consistency in production. Further, while various 3D-printed mechanical devices have been approved by the FDA for clinical use, the incorporation of living cells raises significant safety concerns including the potential for immunogenic reactions, the transmission of diseases, and the long-term viability of the printed tissues [11]. The novel and varied nature of bioprinted products necessitates continuous monitoring for adverse events and long-term outcomes to gather data on the efficacy and safety of these products in real-world settings.

## 7. Conclusions

The exploration of 3D bioprinting in surgery represents a transformative journey from conceptual frameworks to tangible clinical applications across various specialties. This review has showcased the impact and potential of 3D bioprinting technologies in revolutionizing tissue engineering and regenerative medicine, providing innovative solutions for complex surgical challenges. From skin and wound healing to craniofacial, orthopedic, and ophthalmologic reconstructions, the advancements in bioprinting techniques and materials continue to rapidly evolve. Despite the promising developments, several challenges persist, including technical limitations in printing precision, the complexity of integrating multi-tissue systems, and the critical need for vascularization in printed constructs. Moreover, regulatory hurdles continue to be significant barriers that require collaborative efforts between researchers, clinicians, and regulatory bodies to streamline clinical translation.

Future directions should focus on refining bioprinting technologies to enhance the resolution and functionality of bioprinted tissues, exploring novel bioinks that mimic the natural extracellular matrix more closely, and developing more robust in situ bioprinting techniques that could enable surgeries to be more precise and less invasive. The integration of advanced imaging and real-time monitoring within the bioprinting process could further align surgical outcomes with pre-surgical planning, ensuring better patient-specific therapies. As we advance, the dialogue between technological innovation and clinical need will continue to intertwine, paving the way for more sophisticated bioprinting solutions that enhance surgical outcomes in unprecedented ways. The journey of 3D bioprinting from the lab bench to the bedside continues to unfold, promising a future where the regeneration of complex tissues and organs may become routine, significantly improving patient outcomes and quality of life.

## Figures and Tables

**Table 1:** Plastic and reconstructive surgery: representative articles.

Intended Area of Application	Model	Biomaterial	Incorporated Cell Types	3D-Printer Used	Time In Vitro/In Vivo	Variables Assessed	Main Findings	Reference
Skin	Rat	Biodegradable polyurethane (PU) and gelatin hydrogel	Fibroblasts, keratinocytes, and endothelial progenitor cells (EPCs)	Planar-/curvilinear extrusion-based	28 days	Wound closure, re-epithelialization, collagen production, and neovascularization	Successful healing of wounds Complete re-epithelialization Significant collagen production Enhanced neovascularization	[14]
Skin	Mouse	Composite microfragmented adipose extracellular matrix (mFAECM), gelatin methacryloyl (GelMA), and hyaluronic acid methacryloyl (HAMA)	human umbilical vein endothelial cells (HUVECs), fibroblasts, keratinocytes	Extrusion-based	14 days	wound closure rate, collagen deposition, and neovascularization	Cell-laden group exhibited the following: nearly complete wound closure; substantial collagen deposition; pronounced neovascularization as compared to both acellular and negative control groups in all assessed healing metrics	[15]
Skin	Mouse	Alginate hydrogel loaded with nitric oxide (NO) donor	Adipose-derived mesenchymal stem cells (ADSCs)	Extrusion-based	7 and 14 days	Angiogenesis, wound closure rates, epithelialization, and collagen deposition	Cell-laden NO scaffolds significantly enhanced burn wound healing with the following: faster epithelialization; increased collagen deposition; improved neovascularization compared to controls	[16]
Skin	Mouse	Silk fibroin/gelatin hydrogel loaded with methylene blue nanoparticles	N/A	Extrusion-based	1, 3, 7, 10, 14 days	Mechanical strength, biocompatibility, antimicrobial activity, and wound healing effectiveness	Hydrogel demonstrated significant wound closure efficiency and infection management relative to control	[17]
Nasal cartilage	In vitro	GelMA and polycaprolactone (PCL)	Chondrocytes	Extrusion-based	50 days	Cell viability, genotoxicity from ultra-violet light exposure, mechanical properties, and ECM	Viable chondrocyte survival and proliferation with effective ECM secretion Mechanical properties comparable to native nasal cartilage	[18]
Ear cartilage and skin	Immunocompromised rodent	EarSkin: type I collagen hydrogel; EarCartilage: hyaluronan transglutaminase (HATG) based bioink	EarSkin: dermal microvascular endothelial cells and fibroblasts; EarCartilage: auricular chondrocytes	Extrusion-based	28 days	Integration of constructs with host tissue, vascularization, pigmentation, and mechanical stability	Good integration and function, showing effective vascularization and stable mechanical properties	[19]
Trachea	Sheep	PCL	Mesenchymal stem cells (MSCs)	Extrusion-based	21, 42, and 84 days	Integration of material, growth of respiratory epithelium, post-operative recovery, and complications	Two animals showed complete integration with growth of respiratory epithelium; however, two others had poor post-operative recovery and one developed a wound abscess. The study noted the stiffness of the PCL as a limitation	[20]
Trachea	Mice and rabbit	Alternating photocrosslinkable cartilage-specific bioink and vascularized fibrous tissue-specific bioink rings	Chondrocytes and fibroblasts	Extrusion-based	N/A	Integration of material, cellular viability, mechanical properties, tissue-specific regeneration, and epithelialization	Construct successfully mimicked native trachea tissue architecture, showing the following: good integration; mechanical function; promotion of tissue-specific regeneration with epithelialization	[21]
Microvascular tissue	In vitro	Gelatin methacryloyl-sodium alginate hydrogel with fugitive inks for microchannel creation	Endothelial cells and pericytes	Novel extrusion-based printing with dynamic mid-extrusion control	N/A	Cell viability, channel perfusability, hydrogel sheet integrity, and microvascular network formation	High cell viability Effective channel perfusion	[22]
Peripheral nerve	Rat	Decellularized extracellular matrix (dECM) with PCL conduit	N/A	Extrusion-based	N/A	Regeneration efficacy including number of regenerated axons and muscle weight ratio	The 3D-printed construct showed results comparable to autologous nerve grafts and superior to porcine decellularized nerve grafts	[23]

**Table 2:** Orthopedic surgery: representative articles.

Intended Area of Application	Model	Biomaterial	Incorporated Cell Types	3D Printing Technique	Time In Vitro/In Vivo	Variables Assessed	Significant Findings	Reference
Cartilage	In vitro	Chondroitin sulfate (CS) and dermatan sulfate (DS) nanocellulose–alginate	Murine MSCs differentiated into chondrocytes	Extrusion-based	21 days	Compression Young’s modulus; in vitro cytotoxicity; swelling behavior and degradation of scaffold; reverse-transcription polymerase chain reaction (RT-PCR) expression of Collagen (COL)1, COL2, and SRY-Box Transcription Factor 9 (SOX9)	Addition of CS to the bioink showed better results than DS in terms of rheological properties and mechanical properties. However, the DS bioink showed greater metabolic activity and a more typical cartilaginous gene expression profile.	[76]
Cartilage	In vitro	Pentanoate-modified, solubilized, devitalized cartilage hydrogel (PSDVC)	Porcine cartilage and human bone marrow-derived MSCs	Mechanical Dispense	8 days	Hydrogel yield stress, stiffness, swelling behavior, and stress relaxation, and crosslinking time; cell viability	PSDVC hydrogel had the most promising biomechanical performance with high compressive elastic modulus and fast crosslinking (1.7–3 min). PSDVC had high viability of human bone marrow-derived mesenchymal stem cells (hBMSCs) after printing and the paste-like rheological performance provided surgical advantage.	[77]
Bone	In vitro	Alginate–calcium chloride (CaCl_2_), alginate–sulphate (CaSO_4_), alginate–gelatin, and alginate–nanocellulose	Bone marrow-derived MSCs	Extrusion-based	7 days	Material viscosity, cell viability and morphology, anatomical accuracy	Physical properties, stromal cell viability, spreading, and osteogenic potential are all dependent on bioink type.	[78]
Bone	In vitro	Nanocellulose–alginate with hydroxyapatite (HA) and graphene oxide (GO)	Murine MSCs	Extrusion-based	21 days	Bioink viscosity and elastic modulus; cytotoxicity analysis; scaffold swelling, mechanical properties, and degradation; cell viability, metabolic activity, and erythropoietin (EPO) secretion; osteogenic differentiation and mineralization	The addition of HA and GO allowed better rheological property maintenance following sterilization. GO scaffolds showed higher swelling capacity and higher expression of osteogenic markers whereas the addition of HA and GO increased scaffold stability and mechanical properties.	[79]
Cartilage	In vitro	Methacrylated porcine cartilage ECM-based hydrogel	Bone marrow MSCs	Extrusion-based	21 days	Bioink viscosity and shear rate, cell viability, histological and immunohistochemical analysis, mechanical compression testing	Rheologic properties are dependent on the concentration of ECM; however, these properties are independent of cell concentration. This ink had high cell viability and significant chondrogenesis.	[80]
Cartilage	In vitro	Alginate and waterborne polyurethane	Murine chondrogenic cell line	Extrusion-based	28 days	Bioink viscosity; scaffold mechanical strength, elasticity and moistening; glycosaminoglycan and Deoxyribonucleic acid (DNA) quantification	Higher alginate content resulted in the following: higher viscosity; better maintenance of structural interity; improved cell viability.	[81]
Bone	In vitro	Alginate and gelatin	Human MSCs	Extrusion-based	42 days	Geometrical and mechanical analysis of scaffold, cell viability assay, fluid dynamic simulations, micro-CT monitoring, histological analysis	Providing mechanical stimulation (shear stress) to 3D-printed scaffolds induced the following: higher bone volume; increased mineralization.	[82]
Bone	In vitro	Gelatin and oligo (poly-(ethylene glycol) fumarate) (OPF)	Pre-osteoblast cells	Extrusion-based	7 days	Bioink crosslinkability and printability, electron microscopy, cytotoxicity, cell viability, bone/nerve cell proliferation	OPF hydrogels can be used for extrusion-based bioprinting with gelatin concentrations between 3 and 9%. This method maintains cell viability and proliferation.	[83]
Cartilage	In vitro	Extracellular matrix-functionalized alginate	Bone marrow MSC	Extrusion-based	21 or 42 days	Bioink rheological properties and viscosity; chondrogenesis proliferation, cell viability assay, DNA/glycosaminoglycan/collagen assays, histological analysis, RT-PCR; scaffold mechanical analysis	This bioink was found to enhance chondrogenesis. Depositing this bioink with polycaprolactone produced constructs with native cartilage mechanical properties.	[84]
Cartilage/Bone	Mice, Rat	Fibrinogen, type A gelatin, hyaluronic acid, and glycerol	Bone marrow-derived MSCs	Fused deposition modeling	12 weeks	Histological and immunohistochemical analysis, micro-CT analysis	Chondrogenic priming is necessary to trigger construct mineralization and vascularization. Bone Morphogenic Protein -2 (BMP-2)-loaded collagen sponges had higher mineralization compared to experimental groups; however, they were associated with greater heterotopic bone formation. Endochondral bone formation can be directed via cartilaginous templates.	[85]
Bone	Rat	Superparamagnetic iron oxide nanoparticles (SPION)	Mice embryonic fibroblasts and bone osteoblast-like cells	Mechanical Dispense	2, 8, and 12 weeks	Compression testing, micro-CT analysis, antibacterial activity assays, histological analysis	SPION scaffolds had enhanced bacteriostatic properties with a complex, customizable structure. The scaffold was able to achieve complete bone bridging.	[86]
Cartilage	Rat	Gelatin methacrylate, hyaluronic acid methacrylate, and chondroitin sulfate methacrylate	Synovium-derived MSCs (SMSCs)	Extrusion-based	4 and 12 weeks	Bioink preparation: scaffold morphology, swelling, degradation, mechanical strength, printability analysis; in vitro: biocompatibility, chondrogenic differentiation, and Ribonucleic acid (RNA) sequencing; in vivo: histological, microCT, and gait analysis	This bioink successfully promoted the deposition of cartilage-specific ECM. Transforming Growth Factor Beta 1 (TGF-β1) enhanced the aggregation, proliferation, and differentiation of SMSCs. These scaffolds with TGF-β1 promoted cartilage regeneration and functional recovery in vivo.	[87]
Bone/Cartilage	Rabbit	Anisotropic bicellular living hydrogels (ABLHs)	Articular cartilage progenitor cells (ACPCs) and bone MSCs (BMSCs)	Extrusion-based	6 and 12 weeks	Cell proliferation rate, cell viability, confocal microscopy, gene expression via RT-PCR, histologic and immunohistochemical analysis.	ACPCs and BMSCs were successfully differentiated into chondrocytes and osteoblasts, respectively. Implantation of ABLHs achieved biomimetic remodeling of the osteochondral interface.	[88]
Cartilage	Rat	Fibrin–gelatin–hyaluronic hydrogel	Human umbilical vein endothelial cells (HUVECs) and bone marrow-derived MSCs (BMSCs)	Stereolithography	4 and 8 weeks	Micro-CT, histological analysis	BMSCs can be differentiated into hypertrophic cartilage microtissues, which were successfully mineralized in vivo and enhanced by pre-vascularization.	[89]
Bone	Mice	Gelatin, fibrinogen, hyaluronic acid, glycerol, Pluronic F-127, thrombin, PCL, and bone morphogenetic protein 4 (BMP-4)	Bone marrow-derived MSCs and endothelial progenitor cells (EPCs)	Digital light processing	2 and 4 weeks	Scaffold visualization, porosity, and mechanical analysis and micro-CT; cell viability assay, osteogenic differentiation, osteoblast activity, angiogenesis differentiation; histological analysis and immunohistochemistry	Successful production of bioink-PCL osteon-mimetic scaffold with medullary canals, Haversian canals, and Volkmann canals was achieved. Bone marrow-derived MSCs and EPCs mimicked native cell pattern and induced angiogenesis and osteogenesis, both of which were improved by BMP-4 release.	[90]
Bone	Human	Polypropylene fumarate, free radical polymerized polyethylene glycol-polycaprolactone (PEG-PCL-PEG), and Pluronic PF 127	NA	Stereolithography	NA	Scaffold morphology, matrix strength, and matrix resilience; drug release kinetics	Pseudo-bone drug delivery scaffold was successfully designed to mimic healthy human clavicle while delivering Simvastatin. The scaffold promoted bone adhesion and sustained drug release over 20 days.	[91]
Cartilage	Rabbit	Acellular Wharton’s jelly and gelatin methacrylate	Bone marrow-derived MSCs	Extrusion-based	6 and 12 weeks	Scaffold morphology, proteomic analysis, mechanical analysis, swelling, porosity, and rheological analysis; Collagenase degradation, cell viability, chondrogenesis; in vivo morphology and histological analysis	Successful development of cartilage-mimicking scaffold with promising biocompatibility was achieved. Biological and physicochemical analysis demonstrated an environment suitable for articular cartilage defect repair.	[92]
Bone	Rabbit	Fibrin-based hydrogel with gelatin methacrylate and nano hydroxyapatite-coated PCL	Bone marrow-derived MSCs and human umbilical vein endothelial cells (HUVECs)	Extrusion-based	12 weeks	In vitro: cell viability assay, micro vessel assessment; in vivo: vascular micro-CT, colony-forming unit analysis, and histological analysis	Successful development of pre-vascularized tissues leading to accelerated angiogenesis and early bone formation in a critically sized femoral defect.	[93]
Bone	Mice	PCL with decellularized bone ECM	Bone marrow MSCs	Extrusion-based	2 and 8 weeks	Mechanical analysis; cell viability assay, DNA quantification, calcium deposition, ECM characterization, immunofluorescence analysis, micro-CT imaging, histochemical analysis	Adding decellularized bone matrix to PCL scaffolds enhanced mechanical properties and osteogenesis while promoting the following: cellular attachment; angiogenesis; new bone formation.	[94]

**Table 3:** Ophthalmology: representative studies.

Intended Area of Application	Model	Biomaterial	Incorporated Cell Types	3D Printing Technique	Time In Vitro/In Vivo	Variables Assessed	Significant Findings	Reference
Cornea	In vitro	Sodium alginate, gelatin Type B, and Type I bovine collagen hydrogel matrix	Human corneal keratocyte (HCK) cells	Extrusion-based	14 days	Cell viability, cellular morphology, and scaffold structural integrity	Bioprinted corneas retained both their structure and optical clarity. Most corneal keratinocytes remained viable for two weeks and demonstrated expression of fibronectin and actin green.	[182]
Cornea	In vitro	Gelatin methacryloyl/methylcellulose (GelMA/MC) hydrogels	Goat stromal cells	Pneumatic extrusion-based 3D bioprinter	14 days	Cell viability and proliferation, hydrogel mechanical property, degradation rates, and optical transparency	The corneal hydrogels achieved optimal transparency similar to that of normal corneal tissue. The hydrogels promoted cell proliferation and adhesion. Overall, the model mimicked the biomechanical characteristics of the human cornea.	[183]
Cornea	In vitro	Hyaluronic acid (HA) -based bioink	Human adipose tissue -derived stem cells (hASCs)	Extrusion-based	7 days	scaffold mechanical properties and transparency, cell viability and proliferation, cell morphology and tissue formation, and ex vivo integration on porcine cornea	This demonstrated the efficacy of a multi-material bioprinting strategy. This approach created a cornea with good physicochemical and biological properties. In addition, utilizing the soft and stiff bioinks allowed the development of a graft mimicking the organization of the corneal stroma.	[181]
Retina	Laser induced neovascularization mouse model; induced retinal degeneration mouse model	Retinal decellularized extracellular matrix	Human Muller cells	Extrusion-based	7 days	Bioink properties, cell viability and differentiation, and protective effects in reducing vascular abnormalities and retinal protection	The development of a retinal decellularized extracellular matrix was discussed. The developed bioink showed a greater retinal differentiation tendency than Muller cells in collagen and may be a promising candidate for retinal regeneration.	[184]
Retina	Rabbit	Gelatin methacrylate (GelMa) solution	Retinal pigment epithelial (RPE) cells	Inkjet bioprinting	3 days	Cell viability and proliferation, printability and mechanical stability of bioinks, post-implantation integration, and functionality	A retinal pigment epithelial (RPE) monolayer was produced via inkjet bioprinting, and photoreceptor (PR) cells were deposited on top of the bioprinted layer. Structural components of both cell layers were confirmed and the functional properties of the RPE layer was demonstrated by production of human vascular endothelial growth factors.	[185]
Retina	Rat	Bruchs membrane extracellular matrix	RPE cells	Extrusion-based	21 days	Bioink properties, cell viability and proliferation, barrier function, phagocytosis ability, polarized secretion, and implantation integration	This demonstrated the development a Bruchs membrane-derived bioink with an associated RPE layer. The function of RPE cells using the developed bioink was shown to be enhanced as compared to a conventional laminin bioink.	[186]
Cornea	Rabbit	Decellularized extracellular matrix	Primary human and rabbit corneal fibroblasts	Digital light processing (DLP) 3D bioprinting	14 days	Hydrogel properties, cell viability and proliferation, surgical outcomes, corneal healing	A bioprinted cornea was studied in vivo using an electrospun micro-nanofibrous decellularized extracellular matrix. The cornea demonstrated the following: enhanced mechanical properties; the capacity for regeneration; guidance for cell organization; phenotypic characteristics of keratocytes.	[187]

## Data Availability

No new data were created or analyzed in this study. Data sharing is not applicable to this article.
